# Scalable, Flexible, and Affordable Hybrid IoT-Based Ambient Monitoring Sensor Node with UWB-Based Localization

**DOI:** 10.3390/s25134061

**Published:** 2025-06-29

**Authors:** Mohammed Faeik Ruzaij Al-Okby, Thomas Roddelkopf, Jiahao Huang, Mohsin Bukhari, Kerstin Thurow

**Affiliations:** 1 Center for Life Science Automation (Celisca), University of Rostock, 18119 Rostock, Germany; jiahao.huang@celisca.de (J.H.); kerstin.thurow@celisca.de (K.T.); 2 Technical Institute of Babylon, Al-Furat Al-Awsat Technical University (ATU), Kufa 54003, Iraq; 3 Institute of Automation, University of Rostock, 18119 Rostock, Germany; thomas.roddelkopf@celisca.de (T.R.); mohsin.bukhari@uni-rostock.de (M.B.)

**Keywords:** indoor positioning, object tracking, ultra-wideband, Internet of Things, ambient monitoring, gas detection, sensor node

## Abstract

Ambient monitoring in chemical laboratories and industrial sites that use toxic, hazardous, or flammable materials is essential to protect the lives of workers, material resources, and infrastructure at these sites. In this research paper, we present an innovative approach for developing a low-cost and portable sensor node that detects and warns of hazardous chemical gas and vapor leaks. The system also enables leak location tracking using an indoor tracking and positioning system operating in ultra-wideband (UWB) technology. An array of sensors is used to detect gases, vapors, and airborne particles, while the leak location is identified through a UWB unit integrated with an Internet of Things (IoT) processor. This processor transmits real-time location data and sensor readings via wireless fidelity (Wi-Fi). The real-time indoor positioning system (IPS) can automatically select a tracking area based on the distances measured from the three nearest anchors of the movable sensor node. The environmental sensor data and distances between the node and the anchors are transmitted to the cloud in JSON format via the user datagram protocol (UDP), which allows the fastest possible data rate. A monitoring server was developed in Python to track the movement of the portable sensor node and display live measurements of the environment. The system was tested by selecting different paths between several adjacent areas with a chemical leakage of different volatile organic compounds (VOCs) in the test path. The experimental tests demonstrated good accuracy in both hazardous gas detection and location tracking. The system successfully issued a leak warning for all tested material samples with volumes up to 500 microliters and achieved a positional accuracy of approximately 50 cm under conditions without major obstacles obstructing the UWB signal between the active system units.

## 1. Introduction

Recent advances in communication and electronics, along with rapid development in software, deep learning, and artificial intelligence, are driving the adoption of smart systems across various environments, from homes, schools, and laboratories to factories and industrial sites. These technologies aim to improve the quality of life, enhance comfort, and enable the efficient and accurate completion of everyday tasks. Indoor tracking and positioning systems are widely used in many areas of modern life, including tracking luggage and valuable assets, guiding people to specific locations in large buildings such as airports, hospitals, and shopping malls, as well as monitoring the movement of robots and unmanned vehicles for both private and public applications [1]. The process of developing internal tracking and positioning systems went through many stages, depending on the technology and techniques available at that time. Currently, various techniques are in use that have proven their effectiveness in practical applications. The choice of method depends on the specific requirements of the implementation site, including the cost, accuracy, coverage area, and expected number of objects to be tracked to ensure consistent and reliable tracking. The use of Wi-Fi, Bluetooth, cameras and image processing, UWB, ultrasound, and acoustic systems are among the most prominent technologies used at present. Wi-Fi localization typically achieves an accuracy range of 1 to 10 m, while Bluetooth localization generally offers accuracy between 2 and 5 m. Many researchers have tried to use Wi-Fi technology to implement indoor tracking and positioning systems.

Chan et al. proposed a passive Wi-Fi-based IPS using Wi-Fi sniffers. Wi-Fi sniffers are used to scan the coverage area and find the device under test (movable tags) based on received signal strength indicators (RSSIs) as Wi-Fi fingerprints, which are then drawn concerning the reference points (anchors) in the real world. The positioning algorithm used the weighted k-nearest neighborhood (WKNN) method, and the RSSI data for each anchor were collected using a surveying robot for two Wi-Fi bands, 2.4 and 5 GHz. The experimental results indicate that the system positioning accuracy can reach 2.2 m [2]. Huang et al. proposed an indoor localization method with multiple Wi-Fi assistance based on machine learning. Four Wi-Fi devices were used as system anchors and fixed in the corners of the testing area. Several methods were used to implement the localization system using Wi-Fi signals, such as triangulation based on RSSI, fingerprinting based on KNN, and multi-Wi-Fi-assisted localization based on a backpropagation algorithm. The experimental results prove that the backpropagation (BP) algorithm has the best performance and the highest accuracy and stability, with a cumulative distribution function of approximately 0.92 and a mean square error of 0.84 m [3].

Bluetooth low-energy (BLE) technology has been used as another solution to implement indoor tracking and positioning systems. Yukhimets et al. proposed a BLE-based local navigation system for robots using BLE beacons. The proposed system uses several ESP32 modules as anchors at predefined points and a controller installed in the mobile robot as a tag. The position calculation of the movable tag is calculated based on the RSSI of the system anchors in the coverage area. A neural network-based model was used with experimental data and included in the Levenberg–Marquardt method to calculate the optimal estimation of the robot position and enhance the system accuracy. The system testing results indicate that the navigation errors are approximately ±5 cm [4].

Many researchers have successfully used UWB technology to implement indoor tracking and positioning systems. Al-Okby et al. proposed a UWB-based, multi-tag real-time IPS for object tracking. The system consists of several units of the commercial MaUWB_DW3000 (Makerfabs, Shenzhen, China) UWB modules that can be configured as both tags and anchors. The system used a Python-based (Pycharm, 2024.1) monitoring server for viewing the live tracking data. The trilateration method with the least-squares algorithm was used to calculate the coordinates of the movable tags after feeding the monitoring program with the user-defined anchor position and the automatically generated distances between the UWB units. The system test proved an accuracy of ≈50 cm in line-of-sight conditions [5].

Acoustic and ultrasound technologies have been used by another group of researchers to implement an indoor positioning and object tracking system. Wang et al. proposed an acoustic-based indoor localization system for mobile robots using directional variability. The system consists of several anchors made of a metamaterial structure and a speaker used as a movable tag. The anchors are fixed at defined positions and utilize the phenomenon of acoustic diffraction to embed the direction-specific signatures into the continuously emitted sound signals that cover the localization area. The coordinates of the movable tag (speaker) are calculated by recording the acoustic signal from the fixed anchor to calculate the accurate position. The experimental tests show that the system has a good accuracy of ≈6 cm [6]. Other technologies can be used for implementing an IPS, such as radio frequency identification based (RFID) [7], vision and camera based [8], and a hybrid system [9].

Recent research suggests the use of an augmented reality (AR)-based IPS. Peng et al. introduces an AR-based IPS called “EPAR” (efficient and privacy-aware AR framework), which addresses key challenges such as privacy, scalability, accuracy, and efficiency in indoor localization services. EPAR is the first system to simultaneously tackle these aspects, offering a new approach to protect users’ privacy while maintaining and also improving system performance. The system determines users’ locations incrementally, based on individual privacy preferences. The system has been extensively tested, and the test results revealed a good effectiveness of the system [10]. A similar AR-based system is described in [11].

On the other hand, the leakage of toxic, hazardous, or flammable gases is a common and well-known problem at work sites, which requires many preventative measures and expensive infrastructure to be implemented to avoid its occurrence and its dangerous effects. Many researchers have developed sensor nodes to detect chemical leaks and fires using different types of environmental sensors such as metal–oxide–semiconductor [12], optical [13], electrochemical [14], and hybrid sensors [15].

This paper presents a novel approach to designing a portable sensor node capable of detecting hazardous gases and vapors while precisely tracking its location. The key innovation lies in the positioning system’s ability to dynamically select the three nearest available anchors by measuring distances between the sensor node and a network of fixed anchor using UWB technology. This dynamic anchor selection enhances the system’s scalability and enables coverage of larger tracking areas. The IoT processor facilitates data transfer from various environmental sensors to assess surrounding conditions while also transmitting distance measurements from UWB units. These combined data allow the monitoring server to map the information onto a building layout, enabling real-time tracking of the sensor node’s movement along with immediate display of sensor readings. This system also supports an alerting mechanism in case of any abnormal readings. The subsections that follow explain the most prominent methods used to implement an IPS.

### 1.1. Trilateration

Trilateration is one of the most widely used methods used for implementing indoor tracking and positioning systems. In this work, it was employed to calculate the coordinates of moving tags. Various technologies such as Bluetooth, Wi-Fi, or UWB can be used to perform trilateration. The position of the tracked objects is determined by calculating its geometric distances to at least three reference points (anchors) with known coordinates within the coverage area. When UWB is used, these distances are obtained by converting the time of flight (ToF: the time it takes for a wave to travel from the transmitter to the receiver) into distance, using the known propagation speed of UWB signals, which correspond to the speed of light.

The distance d between two points can be determined using their known coordinates (*x, y*); for instance, the distance between the points (*x*_1_, *y*_1_) and (*x*_2_, *y*_2_) is

(1) $$d=\sqrt{{\left(x_{2}-x_{1}\right)}^{2}+{\left(y_{2}-y_{1}\right)}^{2}}$$

If the tag has coordinates ($x_{t},\;y_{t}$) and the anchor has coordinates ($x_{a},\;y_{a}$), the equation can be rewritten as:

$$d_{a}^{2}={\left(x_{a}-x_{t}\right)}^{2}+{\left(y_{a}-y_{t}\right)}^{2}$$

(2) $$d_{a}^{2}=x_{a}^{2}+x_{t}^{2}-2x_{a}x_{t}+y_{a}^{2}+y_{t}^{2}-2y_{a}y_{t}$$

When a point (*r*) is used as a reference point in the IPS, with its anchor fixed at coordinates ($x_{r},\;y_{r}$), and the remaining anchors are located at ($x_{i},\;y_{i}$), where iii denotes the anchor index, then subtracting *d_r_* from *d_i_* yields:

(3) $$d_{r}^{2}-d_{i\;}^{2}{+x}_{i}^{2}+y_{i}^{2}-x_{r}^{2}-y_{r}^{2}=2(x_{i}-x_{r})x_{t}+2(y_{i}-y_{r})y_{t}$$

With *r* = 1 and *a* = 2, 3, … (where *i* represents all the anchors used), we obtain:

(4) $$\left[\begin{array}{cc} 2(x_{2}-x_{1}) & 2(y_{2}-y_{1})\\ 2(x_{3}-x_{1}) & 2(y_{3}-y_{1}) \end{array}\right]\left[\begin{array}{c} x_{t}\\ y_{t} \end{array}\right]=\left[\begin{array}{c} d_{1}^{2}-d_{2\;}^{2}{+x}_{2}^{2}+y_{2}^{2}-x_{1}^{2}-y_{1}^{2}\\ d_{1}^{2}-d_{3\;}^{2}{+x}_{3}^{2}+y_{3}^{2}-x_{1}^{2}-y_{1}^{2} \end{array}\right]$$

**Figure 1:**
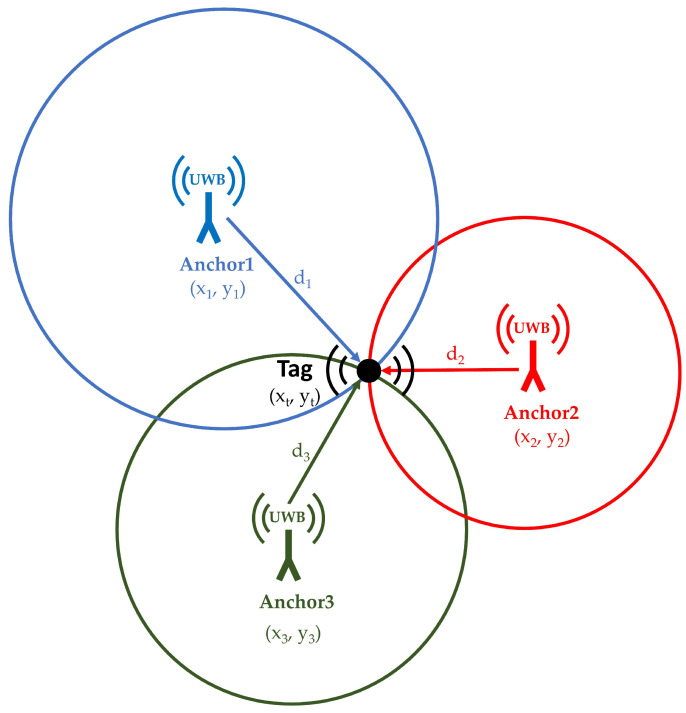
Trilateration technique used for calculating the tag coordinates in an IPS.

By expressing Equation (4) as a linear system in the form *Ax* = *b*, the vector *x* represents the coordinates of the movable tag ($x_{t},\;y_{t}$). These coordinates can then be calculated and monitored using an appropriate positioning algorithm and monitoring software. Figure 1 illustrates the structure of the trilateration technique applied in a three-anchor IPS setup [1].

### 1.2. Triangulation

**Figure 2:**
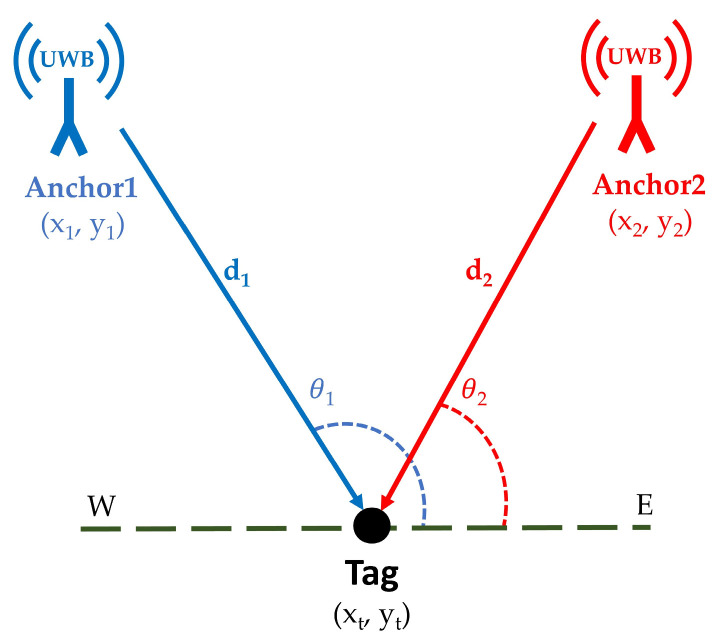
Triangulation-based positioning technique in IPS.

The triangulation technique relies on angle measurements. By knowing the positions of two fixed anchors and estimating the angles between the movable tag and the antennas of these anchors, the position of the tag can be calculated. This is done by extending directional lines from the tag toward the anchors and determining their intersection with the line connecting the two fixed anchors, enabling accurate localization of the tag (see Figure 2).

In Figure 2, the coordinates of anchor1 and anchor2 are ($x_{1},\;y_{1}$) and ($x_{2},\;y_{2}$), respectively, while the coordinates of the movable tag are ($x_{t},\;y_{t}$). Two angles are measured between the anchors, the tag, and the west–east (horizontal) line:

*θ*_1_ = the angle between anchor1 (A1), the tag (T), and E horizontal line (*θ*_1_ = ∠A1TE);

*θ*_2_ = the angle between anchor2 (A2), the tag (T), and E horizontal line (*θ*_2_ = ∠A2TE).

The slope of the line connecting anchor1 and the tag can be calculated using the following formula:

(5) $$tan\theta_{1}=\frac{y_{t}-{\;y}_{1}}{x_{t\;}-x_{1}}$$

Alternatively, the slope of the line connecting anchor2 and the movable tag can also be expressed as:

(6) $$tan\theta_{2}=\frac{y_{t}-y_{2}}{x_{t}-{\;x}_{2}}$$

By solving Equations (5) and (6), we get

(7) $$x_{t}=\frac{\left(y_{1}-y_{2}\right)+x_{2}tan\theta_{2}-x_{1}tan\theta_{1}}{tan\theta_{2}-tan\theta_{1}}$$

and

(8) $$y_{t}=\frac{\left[y_{1}tan\theta_{2\;}-{\;y}_{2}tan\theta_{1}\right]-\left[{(x}_{1}-x_{2})tan\theta_{2}tan\theta_{1}\right]}{tan\theta_{2}-tan\theta_{1}}$$

where *x_t_*, and *y_t_* in Equations (7) and (8) are the estimated movable tag coordinates.

### 1.3. Received Signal Strength Indicator (RSSI)

RSSI is a commonly used method in indoor positioning systems. It measures the power level of a received signal—typically from UWB, Wi-Fi, Bluetooth, or other radio frequency sources—expressed in decibels (dB), to estimate the distance between a transmitter (fixed anchor) and a receiver (movable tag). The underlying principle is that signal strength decreases as the distance increases, allowing positioning algorithms such as multilateration to infer the tag’s relative position by converting RSSI values into distance estimates from multiple anchors.

However, RSSI-based positioning is highly sensitive to obstacles between anchors and the moving tag. Thes obstacles can attenuate or scatter the signal, leading to deviations in the measured signal strength and, consequently, inaccuracies in distance estimation. Such distortions can result in significant positioning errors. Nevertheless, RSSI remains a popular approach due to its simplicity, low implementation cost, and compatibility with standard wireless hardware, making it an attractive and practical choice for many real-time IPS applications.

## 2. Materials and Methods

**Figure 3:**
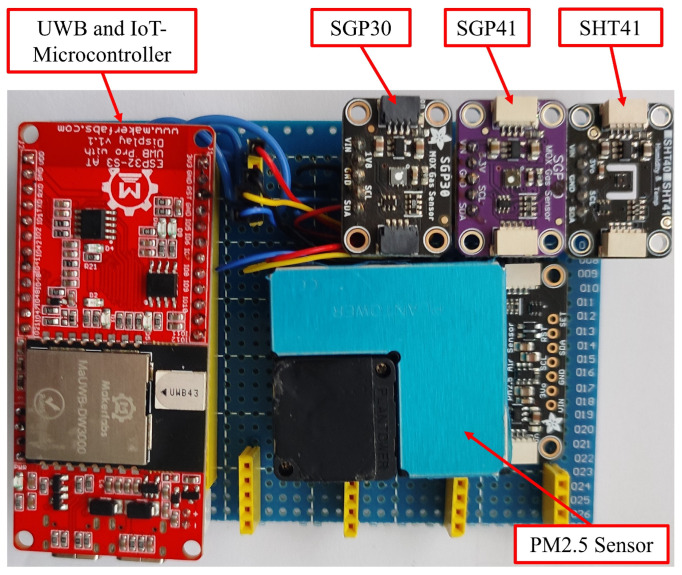
Top view of the sensor node, showing the components used.

In this section, we provide a detailed review of the system’s hardware and software components, covering their characteristics, technical specifications, functionality, and the methods used for data transfer and exchange. All components of the system are located in the mobile sensor node, except for the anchors and the monitoring server. Figure 3 illustrates the components of the mobile sensor node used.

### 2.1. SGP30 Gas Sensor

The SGP30 (Sensirion AG, Stafa, Switzerland) is a metal–oxide–semiconductor (MOX) gas sensor used to measure the concentration of VOCs in the air and provides concentration measurements in parts per billion. This sensor is also used to measure the equivalent concentration of carbon dioxide in the air and provides measurements in parts per million. These measurements are essential for determining the extent of air pollution in the event of a hazardous material leak, fire, or high smoke concentration. The sensor can be integrated with any host processor using the inter-integrated circuit bus (I2C) [16].

### 2.2. SGP41 Gas Sensor

The SGP41 (Sensirion AG, Stafa, Switzerland) is a MOX gas sensor used to measure the VOC index in air and the nitrogen oxide gas index in air (NOx index). The VOC index has a unitless range from 0 to 500, which refers to the air quality in the tested environments, from the best air quality condition (0 to 100) to the moderate (101–250) and up to the hazardous and toxic level (251–500). The NOx index also has a unitless range from 0 up to 500, where the NOx index in normal indoor environments is almost ≈1. In case of fire or pollution of NOx oxides, the index will start increasing up to 500. An alarm threshold of NOx index = 10 can be used to trigger a pollution alarm in the presented monitoring server. The sensor can be integrated with any host processor using the inter-integrated circuit bus (I2C). Additional information can be found in [17].

### 2.3. SHT41 Environmental Sensor

The SHT41 (Sensirion AG, Stafa, Switzerland) is a digital environmental sensor used to measure the temperature in the operation range of −40 to 125 °C and the relative humidity in the operation range of 0 to 100% RH. The use of this sensor is essential for providing the temperature and humidity values to the gas index algorithm of the SGP41 gas sensor. The sensor can be integrated with any host processor using the inter-integrated circuit bus (I2C). More details can be found in [18].

### 2.4. PMSA003I Particulate Matter Sensor

The PMSA003 (Nanchang Panteng Technology Co., Ltd., Nanchang, China) is a versatile digital particle concentration sensor designed to measure the number of suspended particles in the air and provide concentration data through a digital I2C interface. It can be integrated into various instruments and processors related to air quality monitoring or environmental improvement equipment, ensuring accurate and timely particle concentration readings. The sensor offers three particle size measurements: PM1 for particles with a 1-micron diameter, PM2.5 for the commonly monitored 2.5-micron particles, and PM10 for solid particles measuring 10 microns in diameter in the air. This sensor operates based on the laser scattering principle. A laser beam is used to illuminate suspended particles in the air, causing them to scatter light. The scattered light is then collected at a specific angle, and its variations over time are analyzed. Finally, a microprocessor processes these data to determine the equivalent particle diameter and calculate the number of particles of different sizes per unit volume. In this work, measurements from this sensor are used to detect and warn of fire hazards due to its high sensitivity to smoke and airborne solid particles typically released during fire outbreaks [19]. Table 1 provides details of the environmental sensors used and their general specifications.

### 2.5. MaUWB_ESP32S3 UWB Module

UWB technology has existed for decades, but recent advancements in indoor positioning have brought it back into focus. Companies like Decawave (now part of Qorvo Inc.) have played a key role in this resurgence. Their DWM1000 transceiver series has been widely used in real-time indoor tracking and positioning systems.

This work utilizes Decawave’s latest development, the DWM3000 transceiver module (Qorvo Inc., Greensboro, NC, USA), an upgraded version of the DWM1000. The module is built on the Qorvo DW3110 IC and complies with the IEEE 802.15.4-2011 and IEEE 802.15.4z UWB standards. It integrates a ceramic antenna, clock circuit, power management, and essential RF components into a single unit. The module supports two-way ranging (TWR) and time difference of arrival (TDoA) algorithms, making it suitable for real-time tracking and positioning applications. It can also be integrated with any host processor for IPS implementations, and several commercial products based on the DWM3000 are available.

For this implementation, the MaUWB_ESP32S3 module (Makerfabs, Shenzhen, China) with the STM32 AT command was selected. This module includes two microcontrollers:

- STM32F103RCT6 (STMicroelectronics, Geneva, Switzerland): primarily used to simplify the internal configuration of the DWM3000 IC registers;
- ESP32-S3 (Espressif Systems, Shanghai, China): an IoT-based microcontroller with Wi-Fi and Bluetooth capabilities that receives UWB data via AT commands and forwards it to the position-tracking monitoring server of the IPS.

This unit is designed to calculate the distances automatically between the broadband units used in the system using the two-way ranging algorithm. Each tag receives a distance matrix with anchors within the coverage area (maximum eight anchors) and transmits these distances to the monitoring server. In the current work, the ESP32S3 IoT processor was used to transmit distance information, in addition to information measured by a set of environmental sensors, over a Wi-Fi network [20].

### 2.6. Monitoring Server

The monitoring server in indoor tracking and positioning systems receives location data from the IPS via a direct wired connection to a reference anchor or via a wireless network such as Wi-Fi, Bluetooth, or UWB.

The monitoring server then analyzes, sorts, and processes the received data to determine the locations of objects to be tracked in real-time using positioning algorithms such as trilateration, triangulation, and others. It also improves tracking quality by employing specialized algorithms such as least squares, Kalman filtering, and particle filtering. It then projects the tracking coordinates onto real-time building maps through a user interface.

**Figure 4:**
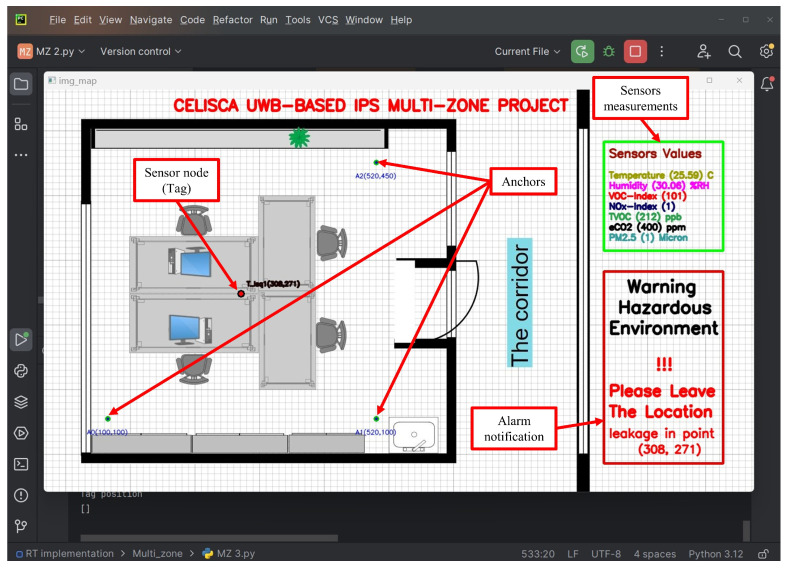
A screenshot of the schematic layout of the monitoring environment based on Python.

In this work, a monitoring and tracking program was developed using Python. The locations of the fixed anchors used in the system are fed directly by the user before the tracking system is activated. The monitoring server receives location and other data from the sensor node (tag) via the Wi-Fi network on the user’s computer. It then sorts the received information into two matrices: the first for calculating the location and the second for displaying data from the various environmental sensors. Trilateration and least-squares (LS) algorithms are used to determine the sensor node’s real-time location coordinates, while a set of environmental data—chosen by the user—is simultaneously displayed. All data can be displayed as needed. The monitoring server displays warning messages whenever the readings exceed acceptable limits for chemical gas concentrations or air quality, alerting to potential leaks or signs of smoke or fire within the building. The monitoring server saves a copy of the processed data as a text file, and the data can also be stored in the main database. Figure 4 shows a screenshot of the Python-based monitoring program.

## 3. Work Description

This work involves implementing two main, complementary tasks. The first task is ambient monitoring, which involves measuring environmental parameters such as the temperature, relative humidity, air quality levels, and particulate matter, and concentrations of certain gases such as carbon dioxide, nitrogen oxides, and VOCs. This task is essential and indispensable for maintaining occupational safety in the workplace. Monitoring environmental factors and using monitoring and warning algorithms provides a significant opportunity to avoid serious accidents such as suffocation, chemical poisoning, or fires and explosions resulting from chemical leaks, which can cause loss of life and equipment.

The second task is to determine the location coordinates within the work area inside the building and in the indoor environments. In the current work, UWB technology was used to accomplish this task. The current approach features a high degree of dynamics in moving between different zones in the coverage area. The tracking and positioning process is implemented using a trilateration algorithm, combined with the least-squares method to minimize errors caused by multipath fading, which occurs when transmission waves arrive via multiple paths. The implementation of the trilateration algorithm requires at least three anchors. The uniqueness of the current work lies in the ability to select the most appropriate anchors for the zone based on the short distance between the moving tag and the adjacent anchors within the coverage area. The tag uses two-way ranging to read the distances to the nearest eight anchors and then selects the closest three anchors to determine its precise location using a trilateration algorithm. All data (environmental and distance data between UWB units) are sent from the sensor node as a single packet using the JSON format. The package includes three types of data: the first is the sensor node’s identification number (tag ID), the second is a distance matrix for eight neighboring anchors, and the third is a data matrix for the environmental sensors. The data are sent from the sensor node using the UDP protocol. The system’s operating steps can be summarized as follows:

- Distribute the anchors to the zones through which the test path passes (four zones), selecting a point to determine the coordinates.
- Measure the x- and y-axes of all installed anchors using a measuring tape and provide the monitoring program with the actual measured coordinates of the installed anchors on the map.
- Operate the mobile sensor node (tag) using a lithium-ion battery and verify the operation of all environmental sensors and the integrated UWB unit by examining the transmitted data using a UDP socket.
- Run the monitoring program.

**Figure 5:**
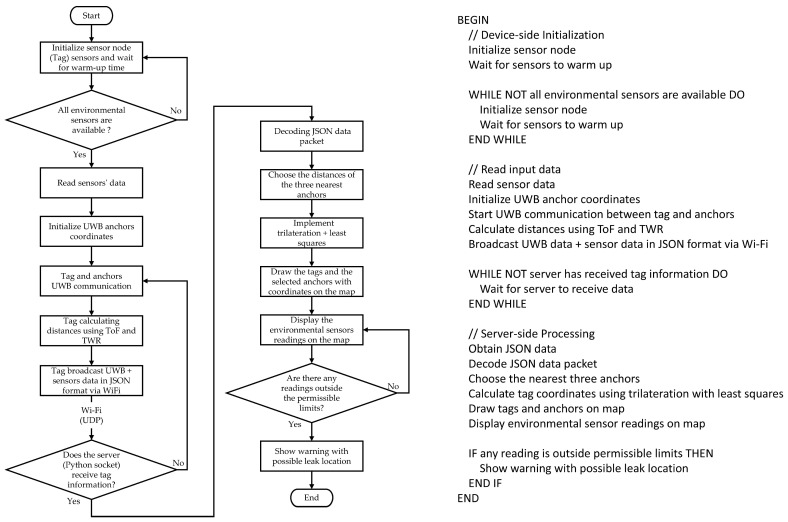
Flowchart and pseudocode for the system operation steps.

In addition to the above, the monitoring program provides a recording of all received data for both site and environmental readings in a text file. A copy of the data can also be stored in the organization’s database. Figure 5 shows a flowchart with a pseudocode of the system’s working steps.

## 4. Experimental Tests and Results

**Figure 6:**
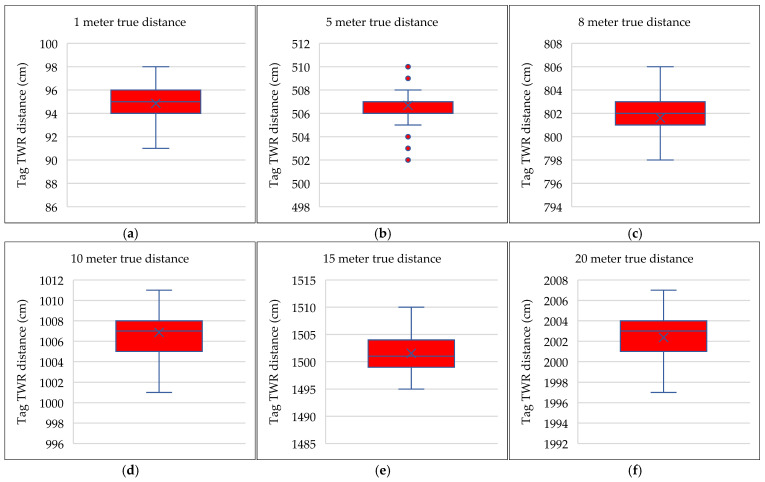
Box and whisker plot for a set of ≈1000 distance measurements between the two UWB units after calibration compared to the actual distances for 1, 5, 8, 10, 15, and 20 m. The y-axis represents the set of readings for the distances measured using the TWR technique between two UWB units after calibration. The margin of error in most readings does not exceed 10 cm.

In preparation for the practical experiments, all units used in the experiments were calibrated to control the delay time resulting from the signal passing through the transmitting antenna. A series of tests were then conducted to assess the accuracy of the units’ TWR distance measurements under line-of-sight conditions, comparing them to actual distances measured manually between two fixed points on the ground using a measuring tape. For this test, distances of 1, 5, 8, 10, 15, and 20 m were selected. The recorded results were consistent across all units, with a margin of error not exceeding 10 cm, except in rare cases, as illustrated in Figure 6.

**Figure 7:**
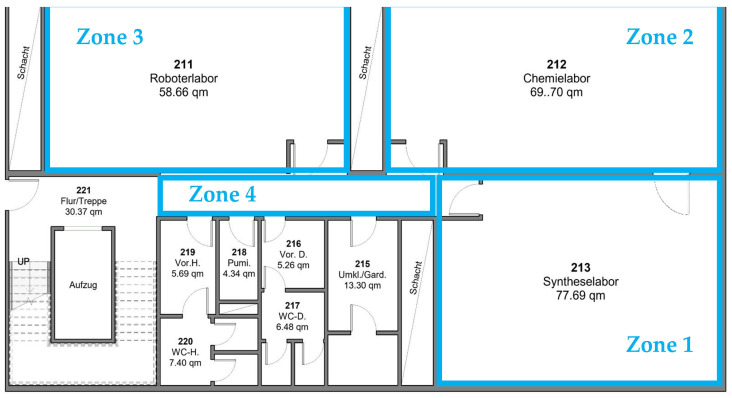
Test area map with the selected zones (1–4).

The system testing process consists of two parts: The first focuses on evaluating the response of the environmental sensors to gas and chemical vapor leaks as the sensor node moves through different zones within the test area. The second part assesses the efficiency and accuracy of the tracking and internal positioning system, with particular emphasis on correctly identifying the leak location during movement. The test area was selected to contain four separate zones, which are groups of automated laboratories separated by a corridor. The tests were conducted in several scenarios that included variations in the test path as well as in the selection of fixed anchor locations in each test. Figure 7 shows the map of the test area with the selected zones (1–4).

### 4.1. Environmental Sensors’ Responses for Selected VOCs

**Figure 8:**
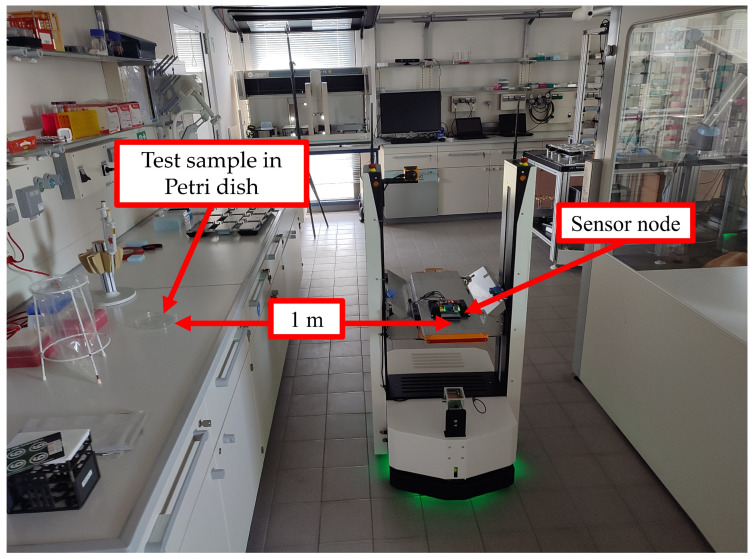
The used ASTI ProBOT L mobile robot.

The main objective of the current work is to provide adequate occupational protection for workers in automated chemical laboratories in the event of a leak of hazardous chemicals or gases. To this end, tests were conducted on a pre-prepared path passing through a series of automated laboratories. The sensor node was installed on an ASTI ProBOT L (ASTI Mobile Robotics, Burgos, Spain) mobile robot (see Figure 8), which was used as a host for the sensor node. The environmental sensors integrated into the current system were carefully chosen based on prior testing detailed in the referenced studies in [21,22].

**Figure 9:**
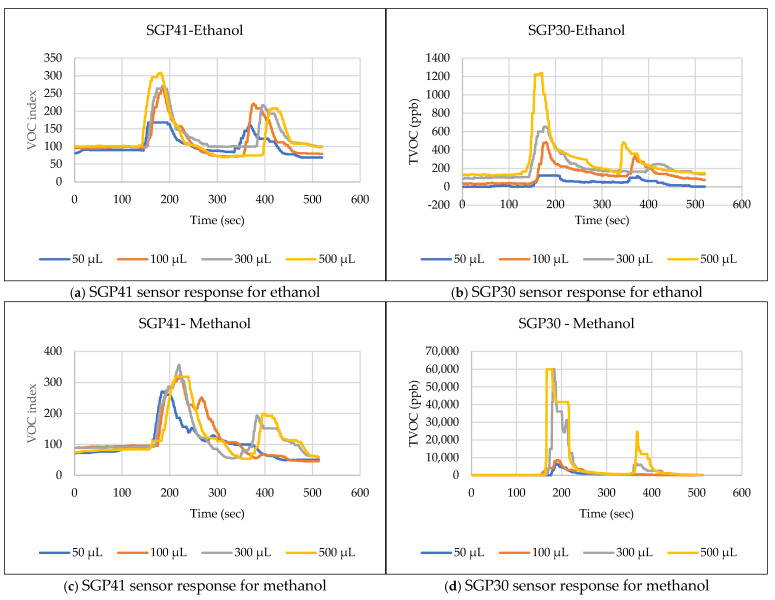

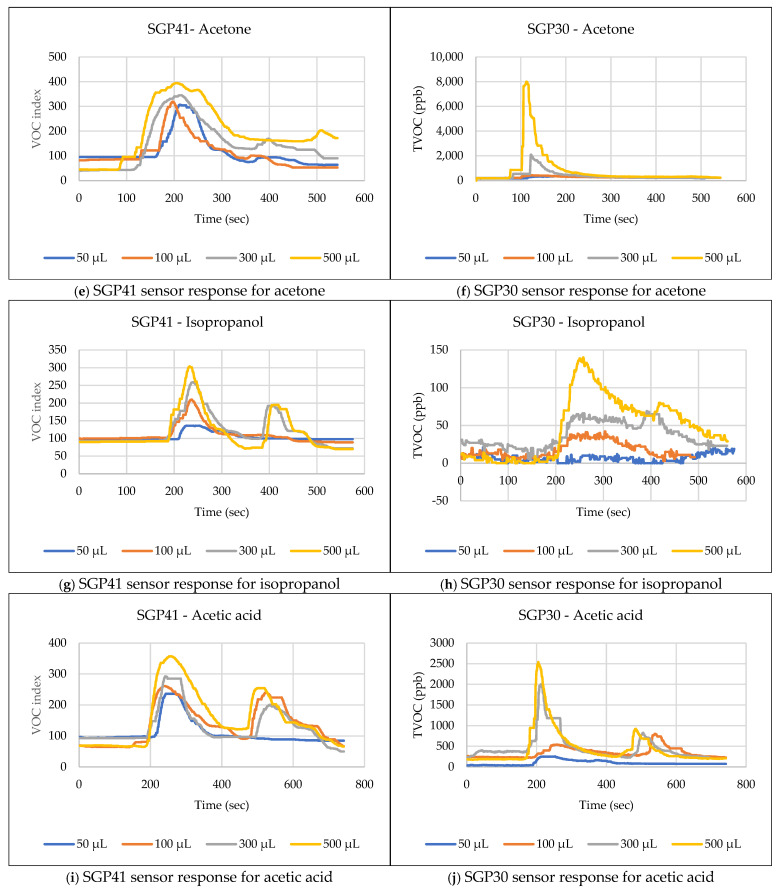

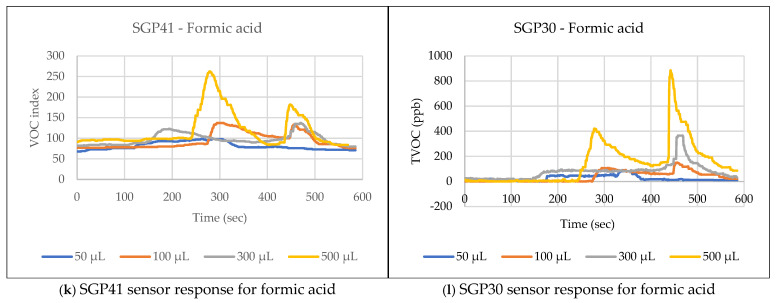
(**a**–**l**) SGP41 and SGP30 sensors’ responses for the selected VOCs.

The test route was chosen to test the response efficiency of environmental sensors within a laboratory. It involved starting from a defined point, passing near the leak area, exiting the laboratory into a leak-free corridor, and then returning to the starting point, passing through the leak area again. Six commonly used VOCs and acids were selected for these experiments, which are ethanol, methanol, acetone, formic acid, acetic acid, and isopropanol. The test was designed to evaluate different sample volumes—50, 100, 300, and 500 µL—to assess the system’s leak detection capability and determine the minimum volume that could be reliably identified. The goal was to ensure that the sensor response would reach a sufficiently high threshold to trigger alarms, prompting worker evacuation or automated actions such as ventilation or fire suppression. The required volume of the test material is injected into a 15 cm diameter Petri dish located approximately 100 cm away from the mobile robot path. The selected volume is injected five seconds before the mobile robot reaches the leak site to allow the test material to change from a liquid to a gaseous state and spread in the area designated for the mobile robot to pass through. Figure 9 explains the responses of the environmental sensors SGP41 and SGP30 for the selected test materials and chosen volumes.

The chemical leakage warning and alert thresholds were selected at 150 (indicates unhealthy air quality) out of the maximum measurement range of 500 for the SGP41 VOC and chemical vapor sensor. The nitrogen oxide threshold was set at 6 (indicates unhealthy air quality) out of the maximum measurement range of 500. The SGP30 VOC threshold was selected at 670 ppb (indicates unhealthy air quality) out of the sensor’s maximum measurement range of 60,000 ppb. The PM2.5 sensor also had a threshold of 10 micrograms per cubic meter. These thresholds were selected roughly in accordance with the relevant environmental regulations for each measured parameter [23]. When the sensor readings exceed predefined limits, an evacuation warning is triggered, and the location of the robot or the person carrying the sensor node is identified using the indoor tracking and positioning system.

**Figure 10:**
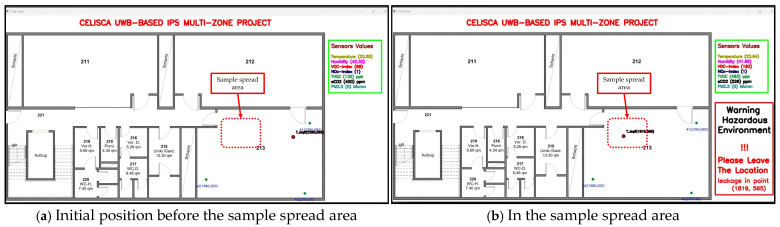

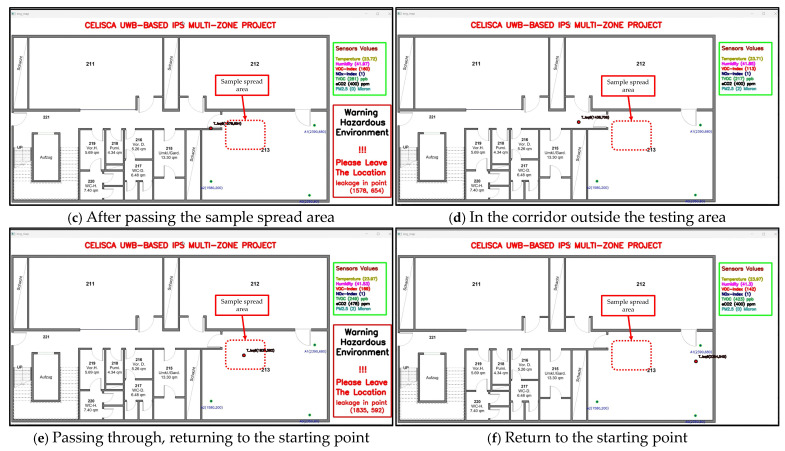
A set of screenshots showing the response of the environmental sensors during the robot’s launch into a laboratory, its passage through a 500 µL ethanol leak, its exit to an external corridor, and its return to the starting point through the same path.

The monitoring program displays the current path of the sensor node along with the data recorded from the various environmental sensors. If the environmental factor readings exceed the user-set limits and thresholds for alarm and warning in the monitoring program, a warning of the detected danger is continuously displayed along with location coordinates until the recorded data return to normal values below the alarm threshold values. Figure 10 explains a set of screenshots of the monitoring program as the robot carrying the sensor node passes through the leak area while testing a range of VOCs and chemical acids.

### 4.2. IPS Testing

In the second part of the laboratory experiments, the robot’s path was carefully selected to cover the four zones selected for the experiments to evaluate the accuracy of the indoor tracking and positioning system operating with UWB technology. The test includes conducting several experiments and testing multiple scenarios using different locations for the anchors used to cover the different zones. In each test, the actual path and its dimensions were measured manually using a measuring tape and then compared with the path recorded by the monitoring program, which was based on UWB distance measurements received from the sensor node. The degree of agreement and deviation between the two paths, as well as the margin of error for each test scenario, was evaluated.

#### 4.2.1. Scenario 1: System Testing in a Single Zone with Three Fixed Anchors

**Figure 11:**
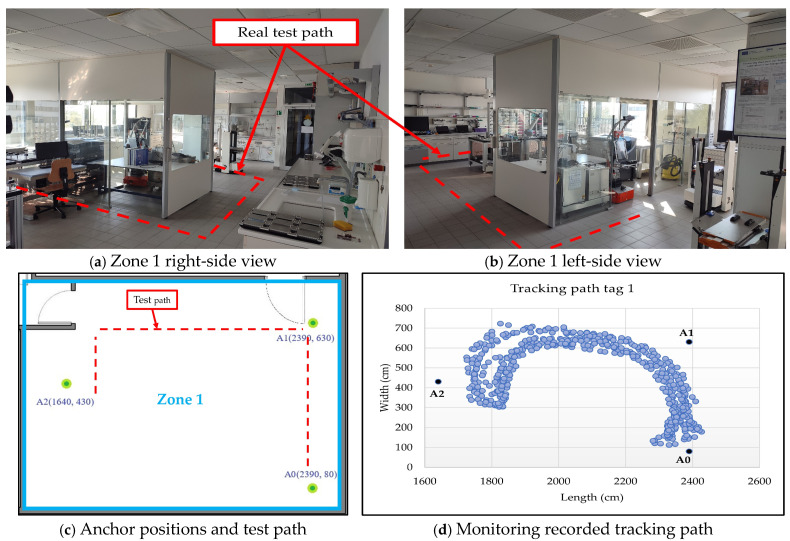
Test environment and scenario 1 for zone 1.

This test is the simplest scenario for testing the accuracy and effectiveness of a system within a single zone. Zone 1 was selected, an automated laboratory containing numerous devices and equipment that may obstruct the line of sight, which is critical for the performance of indoor tracking and positioning systems. Figure 11a,b show the tracking path in real time for zone 1 from different views. Figure 11c shows the anchor positions, and Figure 11d shows the monitoring program’s recorded tracking results.

#### 4.2.2. Scenario 2: System Testing in Two Neighboring Zones with Six Anchors

**Figure 12:**
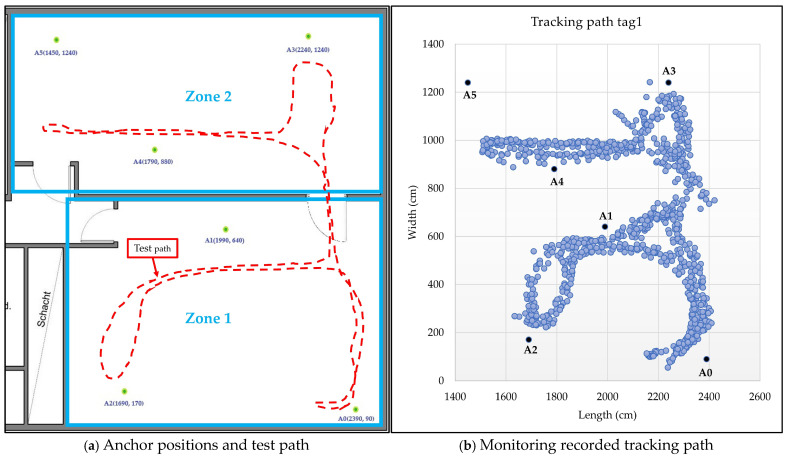
Test scenario 2 for zone 1 and zone 2.

In the second scenario, zones 1 and 2—two adjacent laboratories—were selected for system testing. Both spaces are densely equipped with laboratory devices, with movement largely restricted to narrow corridors. Six anchors (A0–A5) were deployed, with dynamic switching based on their proximity to the mobile sensor node. Figure 12a,b show the tracking path in real time and the recorded tracking path results by the monitoring program.

**Figure 13:**
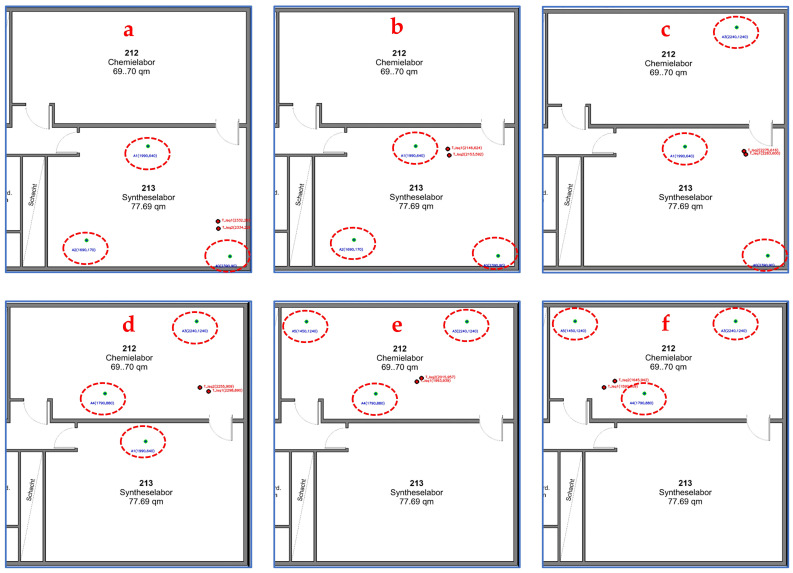
A set of screenshots from the monitoring server showing the dynamic selection process of the nearest three anchors during the execution of the indoor tracking and positioning algorithm. (**a**,**b**) system select anchors A0, A1, and A2; (**c**) system change selected anchors to A0, A1, and A3; (**d**) system change selected anchors to A1, A3, and A4; (**e**,**f**) system change selected anchors to A3, A4, and A5.

Figure 13 illustrates how the system dynamically selects the three anchors closest to the moving sensor node to perform trilateration and apply least-squares algorithms to calculate the coordinates of the moving tags. In Figure 13a, the system selects anchors 0, 1, and 2 to implement the tracking algorithm and continues to do so in Figure 13b. As the moving tags approach the doorway between the two zones (Figure 13c), the system switches to anchors 0, 1, and 3. After entering the second zone (Figure 13d), it updates the selection to the nearest anchors, 1, 4, and 3. Once the tag settles in the center of the second zone, the system selects anchors 3, 4, and 5 for continued tracking (Figure 13e,f). This dynamic anchor-switching mechanism enables continuous and accurate position estimation as the tag moves across multiple zones. Unlike systems that rely on a single fixed reference anchor, this approach significantly enhances scalability and flexibility, allowing coverage over extended or segmented areas.

#### 4.2.3. Scenario 3: System Testing in Two Neighboring Zones with Six Anchors

**Figure 14:**
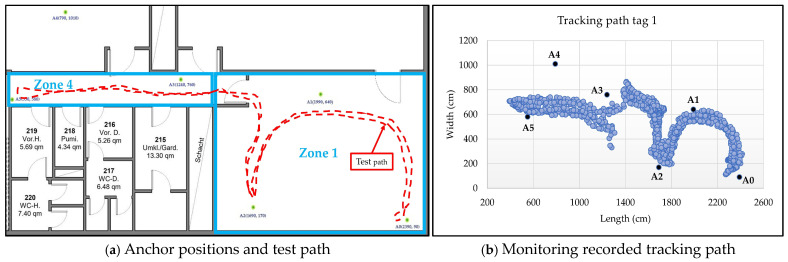
Test scenario 3 for zone 1 and zone 4.

In the third scenario, zones 1 and 4, which are one laboratory and the neighboring corridor, were selected for system testing. In this test, six anchors (A0–A5) were deployed to track the sensor node, with the tracking algorithm dynamically selecting anchors based on their proximity to the moving node. Figure 14a,b show the tracking path in real time and the recorded tracking path results by the monitoring program.

#### 4.2.4. Scenario 4: System Testing in Three Zones with Six Anchors

**Figure 15:**
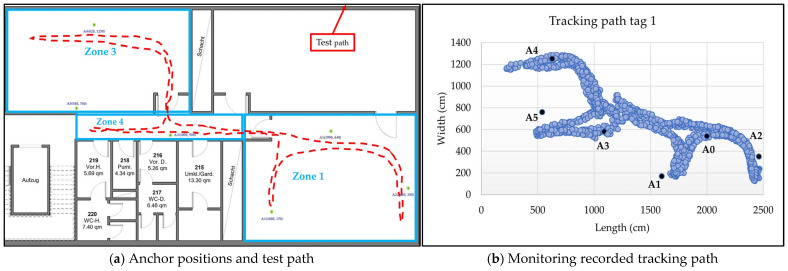
Test scenario 4 for zone 1, zone 3, and zone 4.

In the fourth scenario, the system’s efficiency and accuracy were tested while tracking the sensor node moving from zone 1 to zone 3 via zone 4. Six anchors (A0–A5) were strategically placed to cover the area. Figure 15a,b show the real-time tracking path and the recorded tracking results from the monitoring program.

## 5. Discussion

In this section, we will discuss and analyze the results of the tests in the previous section to evaluate the performance of the system under study. Figure 9 shows the responses of the environmental sensors SGP41 and SGP30 when tested with a range of VOCs and chemical acids. The SGP41 sensor showed excellent responsiveness while passing through the test sample diffusion zone for most materials used in experiments. It provided sufficient response to trigger an alarm for all tested ethanol volumes on both outward and return test paths, recording a maximum VOC index reading of 303. It also showed a good response to methanol for all tested volumes in the outward path and recorded the highest VOC index reading of 357 but failed for smaller samples (50 µL and 100 µL) in the return path. This may be due to the rapid dissipation of this substance in the air in these small samples. The SGP41 sensor showed a strong response to acetone on the outgoing path for all tested volumes, recording a maximum VOC index reading of 393. However, its response was weaker on the return path, failing to detect leaks for the smaller volumes of 50 µL and 100 µL. It was also observed that the warning continued to be triggered even in the leak-free areas for the 500 µL volume, which indicates a high recovery time for this sample. In the isopropanol test, the sensor showed a good response to all samples except the smaller 50 µL sample in the outgoing path and recorded the highest VOC index reading of 301, and the response was lower in the return path, as the sensor failed to detect the two smaller samples, 50 µL and 100 µL. The SGP41 sensor showed a good response to the acetic acid samples, as the sensor was able to detect all samples in the outgoing path and recorded the highest VOC index reading of 356, while the response was lower in the return path, with a failure to detect leakage for the smaller 50 µL sample. The SGP41 sensor showed a weak response to formic acid, detecting only the larger sample volume of 500 µL in both the outbound and return paths, and recorded the highest VOC index reading of 260.

The SGP30 sensor exhibited varied responses to the same previous samples, with some results similar to those of the SGP41 sensor and others differing. For the ethanol sample test, the sensor responded to the samples but did not reach the alarm threshold of 670 ppb except for the largest sample of 500 µL, where the highest reading was 1235 ppb in the outbound path, while the sensor failed to reach the alarm threshold for all samples in the return path and recorded the highest reading of 475 ppb. In the methanol sample tests, the sensor showed better response and detected all the test samples and reached a saturation value of 60,000 ppb (the highest value the sensor can reach within the dynamic range) for the 300 µL and 500 µL samples in the outbound path. On the return path, the responses were weaker but exceeded the threshold for all samples, with the highest reading being 24,690 ppb. In the acetone sample tests, the SGP30 sensor was able to detect only the two larger samples, 300 µL and 500 µL, and the highest reading recorded was 7991 ppb. The sensor failed to detect any sample on the return path. The sensor did not show adequate response to all samples for isopropanol in both runs, and the highest reading recorded was 140 ppb. The sensor showed a different response to acetic acid, reaching the alarm threshold in the outgoing path for both 300 µL and 500 µL samples, with the highest reading recorded being 2484 ppb. The response was weaker during the return path, with the sensor able to reach the alarm threshold for all samples except the smallest sample, 50 µL. Tests of the SGP30 sensor with formic acid samples showed a weak response, as the sensor was unable to reach the threshold for all samples on the outbound path and was only able to reach the threshold for the largest sample of 500 µL on the return path. The highest reading recorded was about 850 ppb.

Figure 10 shows the monitoring program tracking the sensor node’s movement, starting from a point where environmental sensor readings are normal. When the robot carrying the sensor enters the leak area, the program issues a warning about abnormal air quality due to chemical pollutants at the sensor’s location. It also provides the coordinates of the leak area (see Figure 9b,c,e), enabling the observer to take appropriate measures to protect workers, devices, and equipment from potential risk.

Tracking tests of the sensor node demonstrated a high level of dynamism in moving across different coverage areas. The system efficiently selects the three closest anchors based on the sensor node’s location, continuously updating the selections as the node moves relative to the fixed peripheral anchors. The test in Figure 11 shows the system’s performance in a non-line-of-sight area, where an obstacle is located in the center of the area, interrupting the line of sight between the anchors and the moving sensor node. The results indicate a tracking error margin of approximately ±70 cm between the actual path and the path recorded by the monitoring software (see Figure 11d). When coverage was expanded to two adjacent areas separated by concrete walls and glass doors using six anchors (see Figure 12), the system efficiently selected the closest anchors. The largest errors occurred near the obstacle dividing the two areas, with a maximum margin of approximately 90 cm, while the overall error for the test path did not exceed 50 cm. The system performs similarly in scenarios 3 and 4, with error margins close to those in scenario 2 (see Figure 14 and Figure 15). A few anomalous readings exceeded 200 cm due to brief oscillation (<2 s) caused by loss of line of sight, which temporarily led to suboptimal selection of the nearest anchors.

The system was designed with cost efficiency in mind to ensure accessibility and affordability. Each sensor node costs approximately EUR 140, while each fixed anchor, of which at least three are required to cover a single area, costs around EUR 50. The monitoring software was developed using the open-source Python tool, incurring no additional software expenses. To the best of our knowledge, there are currently no commercial systems that combine chemical gas and fire detection with real-time tracking and location, making direct cost comparisons difficult. However, existing indoor positioning and tracking systems are typically expensive and may also require monthly software subscription fees in addition to the initial hardware costs.

## 6. Conclusions and Future Work

In this work, a low-cost mobile sensor node was designed and tested for detecting hazardous gas and chemical vapor leaks in workplace environments. This research presents a comprehensive and cost-effective solution for monitoring industrial areas and scientific laboratory areas containing potentially leaky chemicals by integrating environmental sensors with UWB indoor tracking and positioning systems and IoT capabilities. Data are transmitted directly from the sensor node via Wi-Fi using UDP, ensuring a fast and reliable connection to a cloud-based monitoring server developed in Python. This server displays various environmental parameters alongside the sensor node’s location on a live two-dimensional digital map of the building. Operational tests demonstrated the system’s ability to effectively detect different gas and chemical vapor leaks, tracking leak locations with an average accuracy of approximately 50 cm. Some anomalies reaching up to 200 cm occurred due to the absence of a line of sight between the sensor node and the anchors. The experimental results confirm the system’s effectiveness in detecting various VOC leaks and tracking movement across different zones, making it a promising tool for enhancing safety and operational response in hazardous environments. In future work, additional environmental sensors could be added to broaden the range of detectable hazardous compounds. In addition, integrating inertial sensors, smartphone-based sensing data, augmented reality, advanced deep learning techniques, as well as data fusion and filtering methods such as Kalman filters and particle filters could significantly enhance our approach. These methods can provide valuable contextual information, including spatial layout and user movement patterns, thereby improving the system’s accuracy and reliability, especially in scenarios where the line of sight is obstructed.

## Figures and Tables

**Table 1:** Basic specifications of the used environmental sensors.

Sensor	SGP41 [17]	SGP30 [16]	SHT41 [18]	PMSA003I [19]
Supply (V)	1.7–3.6	1.62–1.98	1.62–3.6	4.5–5.5
Parameters	IAQ Index, NOx Index	TVOC, eCO2	T, RH	PM2.5 (standard), PM1, PM10
Buses	I^2^C	I^2^C	I^2^C	I^2^C
Size (mm^3^)	2.44 × 2.44 × 0.9	2.44 × 2.44 × 0.9	2.0 × 2.0 × 0.75	51.0 × 35.5 × 13.6
Response time	1 s	1 s	4 s	1 s
Structure	MOX	MOX	SMT	digital
Manufacturer	Sensirion AG	Sensirion AG	Sensirion AG	PLANTOWER
I2C address	0 × 59	0 × 58	0 × 44	0 × 12

## Data Availability

The data presented in this work are available on request from the corresponding author.
